# S100A10 silencing suppresses proliferation, migration and invasion of ovarian cancer cells and enhances sensitivity to carboplatin

**DOI:** 10.1186/s13048-019-0592-3

**Published:** 2019-11-18

**Authors:** Lingzhi Wang, Wei Yan, Xukun Li, Zhihua Liu, Tian Tian, Tanxiu Chen, Liang Zou, Zhumei Cui

**Affiliations:** 1 Department of Obstetrics and Gynecology, the Affiliated Hospital of Qingdao University, Qingdao, 266061 People’s Republic of China; 2 Medical Oncology, Jiangxi Cancer Hospital, Nanchang, Jiangxi 330029 People’s Republic of China; 3 State Key Laboratory of Molecular Oncology, National Cancer Center/Cancer Hospital, Chinese Academy of Medical Sciences and Peking Union Medical College, Beiing, 100021 People’s Republic of China; 4 Jiangxi Key Laboratory of Translational Cancer Research, Jiangxi Cancer Hospital, Nanchang, Jiangxi 330029 People’s Republic of China; 5 Department of Science and Education, Jiangxi Cancer Hospital, Nanchang, Jiangxi 330029 People’s Republic of China; 6 Department of anesthesiology, National Cancer Center/Cancer Hospital, Chinese Academy of Medical Sciences and Peking Union Medical College, 17 Panjiayuan Nanli, Chaoyang District, Beijing, 100021 People’s Republic of China

**Keywords:** S100A10, Ovarian cancer, Proliferation, Apoptosis

## Abstract

**Background:**

Ovarian cancer is the leading cause of gynecological cancer-related mortality. The novel oncogene S100A10 has been reported to be involved in cancer cell proliferation, invasion and metastasis. The role of S100A10 in ovarian cancer has not been well studied and the effect of S100A10 on chemotherapy remains unclear. The aims of the present study were to investigate the functional role of S100A10 in the progression and carboplatin sensitivity of ovarian cancer.

**Methods:**

We examined the expression levels in tissues of S100A10 in 138 cases of ovarian cancer by IHC. To determine the functional roles of downregulated S100A10 in ovarian cancer, cell proliferation, colony formation, cell migration and invasion assays were performed. Chemoresistance was analyzed by apoptosis assay. A xenograft tumor model was established to confirm the role of S100A10 in carboplatin resistance in vivo. Using Western blot assays, we also explored the possible mechanisms of S100A10 in ovarian cancer.

**Results:**

The results showed that increased expression of S100A10 was positively associated with carboplatin resistance (*P* < 0.001), tumor grade (*P* = 0.048) and a poorer prognosis (*P* = 0.0053). Functional analyses demonstrated that S100A10 suppression significantly suppressed ovarian cancer cell proliferation, colony formation, cell migration and invasion, remarkably increased carboplatin-induced apoptosis in SKOV3 and A2780 cells and inhibited tumor growth in vivo. Downregulation of S100A10 expression could inhibit cell proliferation and enhance ovarian cancer cell sensitivity to carboplatin, possibly involving the regulation of cleaved-Caspase3 and cleaved-PARP.

**Conclusions:**

Together, the results of the present study reveal that S100A10 expression can be used as a predictive marker for the prognosis of ovarian cancer and chemosensitivity to carboplatin.

## Background

Ovarian cancer is the leading cause of gynecological cancer-related mortality that seriously endanger women’s health [1, 2]. Although there are proven benefits of surgical resection and aggressive treatment with chemo- and radiotherapy, the prognosis of ovarian cancer remains very poor. The high mortality rate is mainly attributed to the late presentation of the disease. Current management strategies include debulking surgery and adjuvant chemotherapy. Among chemotherapies, carboplatin is the most important and basic first-line treatment drug for ovarian cancer. Patients treated with carboplatin usually obtain better results in the initial stage of carboplatin chemotherapy. However, the majority of patients, especially those at an advanced stage, will eventually have a relapse and die of the disease [3, 4]. For the last two decades, there has been no substantial improvement in ovarian cancer survival [5]. Therefore, elucidating the mechanisms involved in the progression and chemotherapy resistance of ovarian cancer is crucial for the discovery of novel molecular prognostic indicators and new therapeutic targets.

S100A10 belongs to the S100 family of low molecular weight (10–12 KD) calcium binding proteins and is usually found in many cell types bound to its ligand annexin A2 as a heterotetrameric (S100A10)_2_-(annexin A2)_2_ complex [6, 7]. A variety of proteins have been reported to interact with S100A10, including miR-590-5p, DLC1, B-FABP and others, indicating that S100A10 is an active regulator that participates in multiple biological functions [8]. In addition, S100A10 has been found to be involved in regulating multiple biological processes, such as cell proliferation, differentiation, apoptosis, inflammation, angiogenesis, motility, migration and invasion. Recently, S100A10 was considered to be an oncogene. For example, elevated levels of S100A10 have been reported to be associated with several types of cancer, including colorectal cancer [9], basal-type breast cancer [10], lung cancer [11], gastric cancer [12] and pancreatic ductal cancer [13]. The role of S100A10 in oxaliplatin sensitivity in human colorectal cancer has also been noted [9]. Noor A. Lokman’s [14] research group demonstrated that the expression of annexin A2 and S100A10 together is a powerful predictor of serous ovarian cancer outcome. However, the functional role of S100A10 in the progression and carboplatin sensitivity of ovarian cancer is currently unknown.

In this study, we initially analyzed the association between S100A10 expression and clinical outcomes of ovarian cancer patients to evaluate the feasibility of S100A10 as a prognostic biomarker. Furthermore, we performed a series of functional assays to determine the role of S100A10 in tumor progression and chemosensitivity of ovarian cancer cells in vitro. In addition, the mechanism of S100A10 in regulating carboplatin sensitivity in ovarian cancer was investigated. We also explored the role of S100A10 in tumor growth in vivo.

## Materials and methods

### Clinical specimens

In this study, clinical samples of 138 patients with ovarian cancer who underwent initial surgical treatment were included and were obtained from the Department of Gynecologic Oncology in the Chinese Academy of Medical Sciences Cancer Hospital (Beijing, China) from May 2007 to January 2013. Each patient gave written informed consent to participate in this research. All experiments conducted using human tissues were approved by the ethics committee of the Chinese Academy of Medical Sciences Cancer Hospital (Beijing, China).

### Immunohistochemistry analysis

Mouse monoclonal antibodies (4E7E10, Novus Biologicals) were used to assess the expression of S100A10 at a dilution of 1: 75. All procedures were performed according to a standard protocol [15] and the manufacturer’s instructions. As a negative control, sections were treated with mouse immunoglobulin G instead of primary antibodies. All slides were scanned by Aperio scanning system (Aperio, San Diego, USA) and the Aperio Image Scope software was employed for quantitative analysis of S100A10 protein expression. Approximately 4 to 6 different parts of the slide were randomly selected for analysis. The intensity score was graded in range from 0 to 3 according to the percentage of positively stained tumor cells. When 0–10% of tumor cells were stained, a score of 0 was given; when 10–25% of tumor cells were stained, a score of 1 was given; when 25–50% of tumor cells were stained, a score of 2 was given; and when 50–100% of tumor cells were stained, a score of 3 was given. The final S100A10 staining score was defined as follows staining score of 0–1 was considered to represent low expression, and 2–3 was considered to represent a high expression. The staining results of S100A10 were evaluated according to the scoring criterion reported in a previous study [16].

### Tumor xenograft assay

Mouse experiments (approval number: SYXK(京)2014–0003) were carried out under the approval of the Chinese Academy of Medical Sciences Cancer Hospital Animal Care and Use Committee, and female NOD/SCID mice at 5–6 weeks of age were purchased from Beijing HFK Bioscience Co. Ltd. and allowed 1 day to adapt. Mice were maintained under specific pathogen-free conditions and provided with normal food and water ad libitum. Tumors were established by subcutaneous injection of 1 × 10^6^ A2780 cells in the right flank of mice. When the maximum diameter of all tumors was close to 1.2 cm, animals were euthanized. The duration of the experiment was 16 days. We used 15 mice per experiment. In each experiment, all animals were euthanized. There were no animals found to be dead. The health and behavior of the animals were monitored every 3 days. Tumor diameter was measured by a caliper. Additionally, only one tumor was found in each mouse.

### Cell lines and cell culture

Ovarian carcinoma cell lines (SKOV3 and A2780) and human embryonic kidney (HEK293T) cells were purchased from the American Tissue Culture Collection (ATCC). RPMI 1640 (Gibco, Thermo Fisher Scientific) with 10% FBS (Gibco, Thermo Fisher Scientific), 100 U/ml penicillin (Life Technologies, Gibco) and 100 mg/ml streptomycin (Life Technologies, Gibco) was used to culture SKOV3 and A2780 cells. HEK293T cells were cultured in Dulbecco’s modified Eagle’s medium (DMEM) supplemented with 10% FBS, 100 U/ml penicillin and 100 mg/ml streptomycin. These cells were maintained at 37 °C in a humidified incubator with 5% CO_2_.

### Construction of plasmids, lentivirus packaging and transfection

For stable knockdown of S100A10, two shRNA sequences (shS100A10–1: CCATGATGTTTACATTTCACA, and shS100A10–2: ACCTGAGAGTACTCATGGAAA) targeting S100A10 were separately inserted into pSIH1-H1-Puro (Invitrogen, Thermo Fisher Scientific, Inc.), and shGFP (Invitrogen, Thermo Fisher Scientific, Inc.) was used as a control. A total of 3 μg of S100A10 silencing or control plasmids were carefully transfected into HEK293T cells together with three packaging plasmids (3 μg PLP1, 3 μg VSVG and 3 μg PLP2) using Lipofectamine 2000 (Invitrogen, CA, USA) with Opti-MEM (Thermo Fisher Scientific). Then, viral supernatant was collected. SKOV3 and A2780 cells (3 × 10^5^) were plated 12 h prior to transfection. Cells were transfected by 200 μl viral supernatant (about 3 × 10^5^ copies/ml lentiviral particles) using Lipofectamine 2000 (Invitrogen, CA, USA) with OPTI-MEM (Thermo Fisher Scientific) according to the manufacturer’s instructions. After 48 h, the supernatant was replaced with fresh medium. Then, stably transduced cells were selected with 1 μg/ml puromycin for 2 weeks. The efficiency of shRNA-mediated silencing of S100A10 was validated by quantitative real-time PCR and Western blot analysis.

### Cell proliferation assay

Cell proliferation was detected by the CCK8 assay at five time points after seeding cells at a density of 1 × 10^3^ cells/well (*n* = 6) in 96-well plates. At the end of the different experimental periods, cells were cultured in 100 μl/well fresh medium mixed with CCK-8 solution (10,1) (Dojindo, Shanghai, China) and incubated for 1 h at 37 °C. Then, OD values were measured at 450 nm using a spectrophotometer (Bio-Rad, Hercules, CA).

### Apoptosis assay

The rate of apoptosis was determined using an Annexin V/PI kit (MultiSciences) according to the manufacturer’s protocol. Briefly, SKOV3 and A2780 cells were incubated with carboplatin or not for 24 h. Subsequently, cells were harvested and resuspended in 500 μl 1X binding buffer. Finally, 5 μl Annexin V-FITC and 10 μl PI were added to the cells for 5 min at room temperature in the dark. Early and late apoptotic cells were analyzed using FACSCalibur (BD Bioscience).

### Quantitative real-time PCR

SKOV3 and A2780 cells were treated as mentioned above. Total RNA was isolated from cells using TRIzol reagent (Invitrogen; Thermo Fisher Scientific, Inc.). Then, the RNA was reverse transcribed to cDNA using a FastQuant RT Kit (TIANGEN Biotech (Beijing) Co. Ltd.). Quantitative real-time PCR was conducted using SYBR Green PCR Master Mix (Applied Biosystems) following standard techniques, as described previously. Next, the 2^-ΔΔCt^ method was used to calculate the relative mRNA expression. The gene-specific primers that were used are listed as follows:

- S100A10-F: AAAGACCCTCTGGCTGTGG
- S100A10-R: AATCCTTCTATGGGGGAAGC
- β-actin-F: CCGTTCCGAAAGTTGCCTTTT;
- β-actin-R: GAGGCGTACAGGGATAGCAC.

### Western blot analysis

For Western blot analysis, total proteins were directly extracted by cell lysis buffer (1% Triton X-100; 10 mM Tris-HCl, pH 7.4; 150 mM NaCl; 0.25% sodium deoxycholate; 5 mM EDTA, pH 7.4). Total proteins (15 μg) were separated by 10–15% SDS-PAGE and transferred onto polyvinylidene difluoride membranes (Millipore, Bedford). After 1 h of incubation in blocking solution the membranes were exposed to primary antibody anti-S100A10 (1:5000, 4E7E10, Novus Biologicals) or anti-β-actin (1:4000, A5316, Sigma) overnight at 4 °C. Following washing in TBS-T 3 times, the respective HRP-linked secondary antibodies were applied at a 1:5000 dilution for 1 h at room temperature. The proteins were finally detected by an enhanced chemiluminescence detection system (Pierce, Rockford, IL, USA).

### Colony formation assay

To detect the effect of S100A10 on colony formation in A2780 cells, we performed colony formation assays. Lentivirus-infected A2780 cells were seeded at a low density (1000 cells/well) in 6-well plates and cultured for 10–14 days. Colonies were stained with 0.1% crystal violet and quantified using ImageJ software (National Institutes of Health, Bethesda, MD).

### Wound-healing, migration and invasion assays

In vitro cell migration ability was assessed by wound-healing assays. When cells were grown to 90% confluency, a straight artificial scratch was created by a 20 μl sterile tip. Then, cell spread across the wound was detected using a phase-contrast microscope at consecutive 48 h time points. For migration assays, SKOV3 and A2780 cells (2 × 10^4^) were plated on membranes with 8.0 μm pores (Corning Incorporated, Corning, NY). For invasion assays, SKOV3 and A2780 cells (2 × 10^4^) were seeded in Matrigel-coated chambers. Cells were resuspended in 100 μl serum-free medium and allowed to invade toward complete growth medium for 24 h. Cells that adhered to the lower surface of the membrane were fixed with cold methanol for 30 min and stained with 0.1% crystal violet for 1 min. After dehydration, migrated or invaded cells were imaged and counted under a light microscope (Leica Microsystems, Mannheim, Germany).

### Statistical analysis

Statistical analyses were performed with SPSS version 13.0 software (NY, USA). Data from three independent experiments are presented as the mean ± standard deviation. To determine the significance of differences among groups, one-way ANOVA followed by Student-Newman-Keuls analysis was performed. The association between S100A10 expression levels and patient clinicopathological factors was analyzed by the χ^2^ test. We utilized the Kaplan-Meier method for evaluating progression-free survival (PFS) and overall survival (OS) rates; the survival curves were compared using the log-rank test. All tests were two sided. The results were considered significant when *P* values were < 0.05.

## Results

### Clinical significance of S100A10 in ovarian cancer tissues

To clarify the biological functions of S100A10 in ovarian cancer, S100A10 expression was immunohistochemically studied in 138 cases of ovarian cancer, and its associations with clinicopathological parameters were evaluated. Kaplan-Meier survival analyses were used to estimate the effect of S100A10 expression on survival. High S100A10 expression levels at age < 60 at diagnosis and tumor grade G3 were significantly higher than those at age ≥ 60 at diagnosis (Table 1). Furthermore, we found that the level of S100A10 upregulation was significantly higher in carboplatin-resistant ovarian cancer (Table 1). However, our data did not show that S100A10 expression was associated with residual tumor size, CA125 levels in primary tumors or FIGO stage (Table 1).

**Table 1:** Distribution of S100A10 status in ovarian cancer according to clinicopathological characteristics

Clinical Variable	Patients	S100A10 expression (No. of cases)		*P* Value
		Low	High	
All cases	138	65	73	
Age at Diagnosis	≥60	24	16	**0.013**
	< 60	45	53	
Residual tumor size (cm)	≥2	19	16	0.557
	< 2	50	53	
Level of CA125 in primary tumors (U/ml)	≥500	50	55	0.318
	< 500	19	14	
Recurrent type	Carboplatin-sensitive	49	26	**< 0.001**
	Carboplatin-resistant	20	43	
FIGO stage	III	57	52	0.296
	IV	12	17	
Tumor grade	G1/G2	29	18	**0.048**
	G3	40	51	

NOTE: *P* value highlighted in bold indicate *P* < *0.05*

**Fig. 1:**
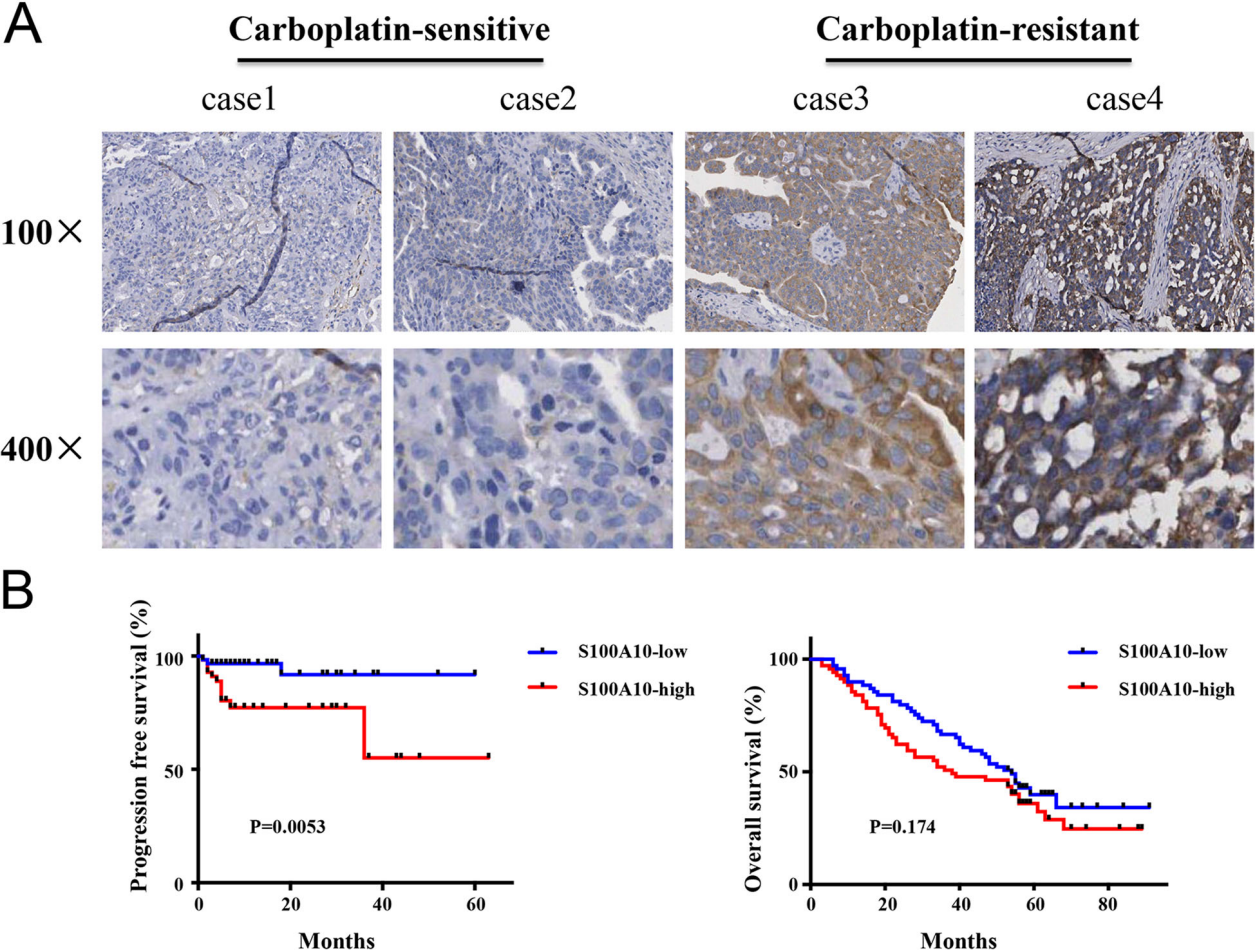
S100A10 was upregulated in ovarian cancer tissues with carboplatin resistance, and high S100A10 expression predicted poor prognosis. (**a**) The expression of S100A10 protein in tumor tissues from patients with carboplatin-sensitive or carboplatin-resistant ovarian cancer by immunohistochemistry analysis and representative staining images are shown. Representative images are shown at × 100 (upper panels) or × 400 (lower panels) magnification. (**b**) Estimation of progression-free survival and overall survival curves by Kaplan-Meier analysis with log-rank test in 138 ovarian cancer patients according to S100A10 expression

S100A10 was mainly localized in the cytoplasmic compartment of ovarian cancer cells (Fig. 1a). In carboplatin-sensitive ovarian cancer tissues, weak cytoplasmic expression of S100A10 was observed (Fig. 1a). In carboplatin-resistant ovarian cancer tissues, strong cytoplasmic expression of S100A10 was observed. The effects of S100A10 protein levels on patient survival were examined with log-rank test. As shown in Fig. 1b, patients with low S100A10 expression tended to have a longer progression-free survival compared with those with high protein expression of S100A10. However, the expression level of S100A10 did not affect overall survival (Fig. 1b). These results demonstrate that S100A10 might function as an oncogene in ovarian cancer progression.

### S100A10 knockdown inhibited cell proliferation and colony formation

**Fig. 2:**
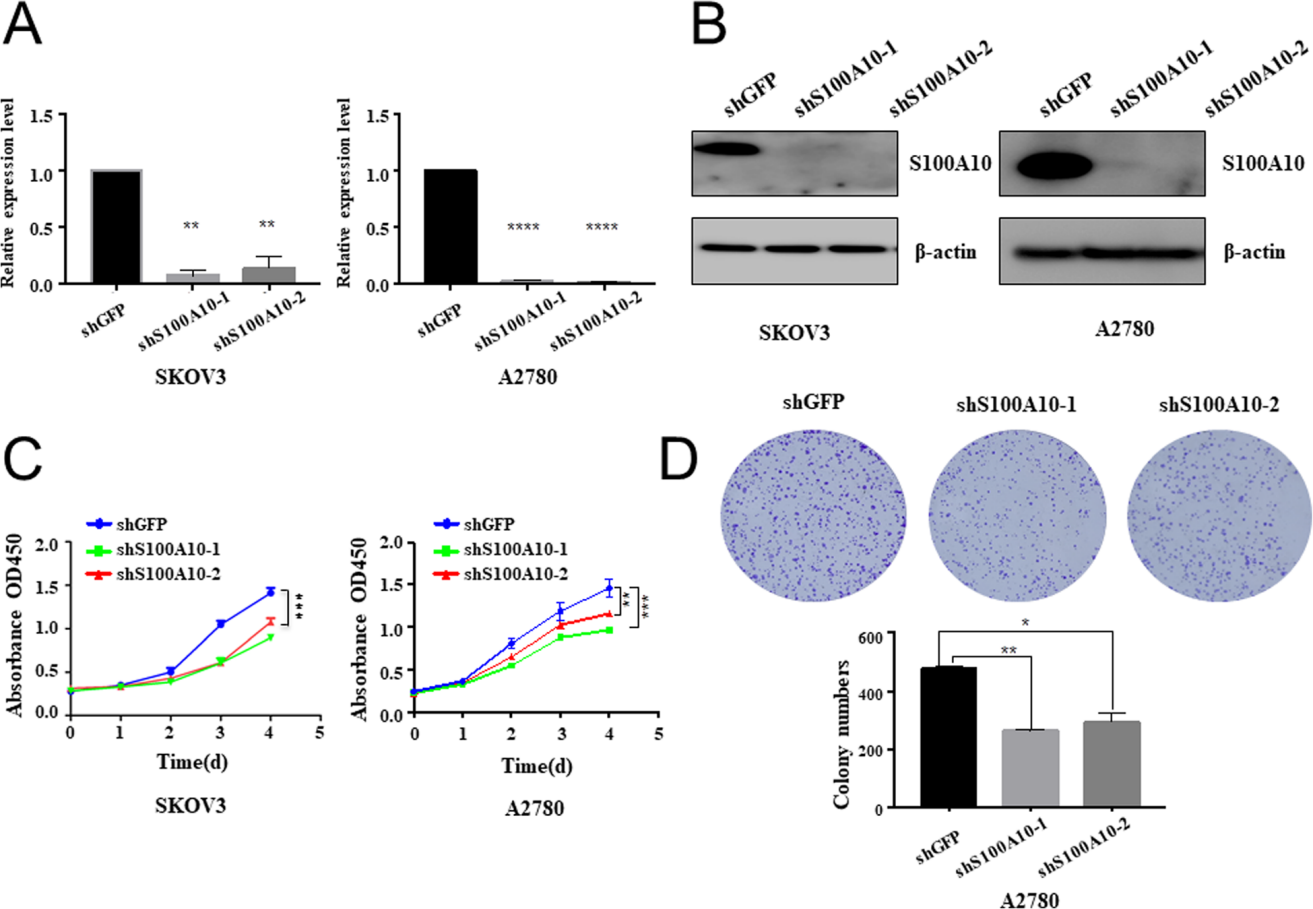
S100A10 knockdown inhibited cell proliferation and colony formation. (**a**) Quantitative real-time PCR showing the expression level of S100A10 mRNA in shGFP, shS100A10–1 or shS100A10–2 SKOV3 and A2780 cell lines. (**b**) Western blots showing the expression of S100A10 protein in shGFP, shS100A10–1 or shS100A10–2 SKOV3 and A2780 cell lines. For quantification, the expression of S100A10 was normalized to that of β-actin. (**c**) CCK-8 assay showing that the knockdown of S100A10 markedly suppressed the proliferation of SKOV3 and A2780 cells. (**d**) Colony formation assay demonstrated that S100A10 knockdown decreased colony numbers. Statistical significance was analyzed by ANOVA. Data are expressed as the mean ± standard deviation (*n* = 3 in each group). * *P* < 0.05, ** *P* < 0.01, *** *P* < 0.001, and **** *P* < 0.0001 vs. the shGFP group

To further investigate the effect of S100A10 on ovarian cancer cell proliferation and colony formation, shRNA-mediated S100A10 knockdown was achieved in SKOV3 and A2780 cells by transfection with shGFP, shS100A10–1 or shS100A10–2. qRT-PCR and Western blot were performed to confirm the transfection efficiency. As shown in Fig. 2a&b, shS100A10–1 and shS100A10–2 both dramatically reduced S100A10 expression in SKOV3 and A2780 cells relative to shGFP-transfected cells. The effect of S100A10 knockdown on the proliferation of SKOV3 and A2780 cells was analyzed. The CCK-8 assay showed that the OD_450_ of SKOV3 and A2780 cells in the shS100A10–1 and shS100A10–2 groups was significantly lower than that in the shGFP group at 96 h (Fig. 2c), suggesting that S100A10 knockdown inhibited the proliferation of SKOV3 and A2780 cells. Knockdown of S100A10 also resulted in the formation of fewer colonies in A2780 cells (Fig. 2d). These results suggested that S100A10 knockdown exhibited a negative effect on ovarian cancer cell proliferation.

### S100A10 knockdown inhibited cell migration and invasion

**Fig. 3:**
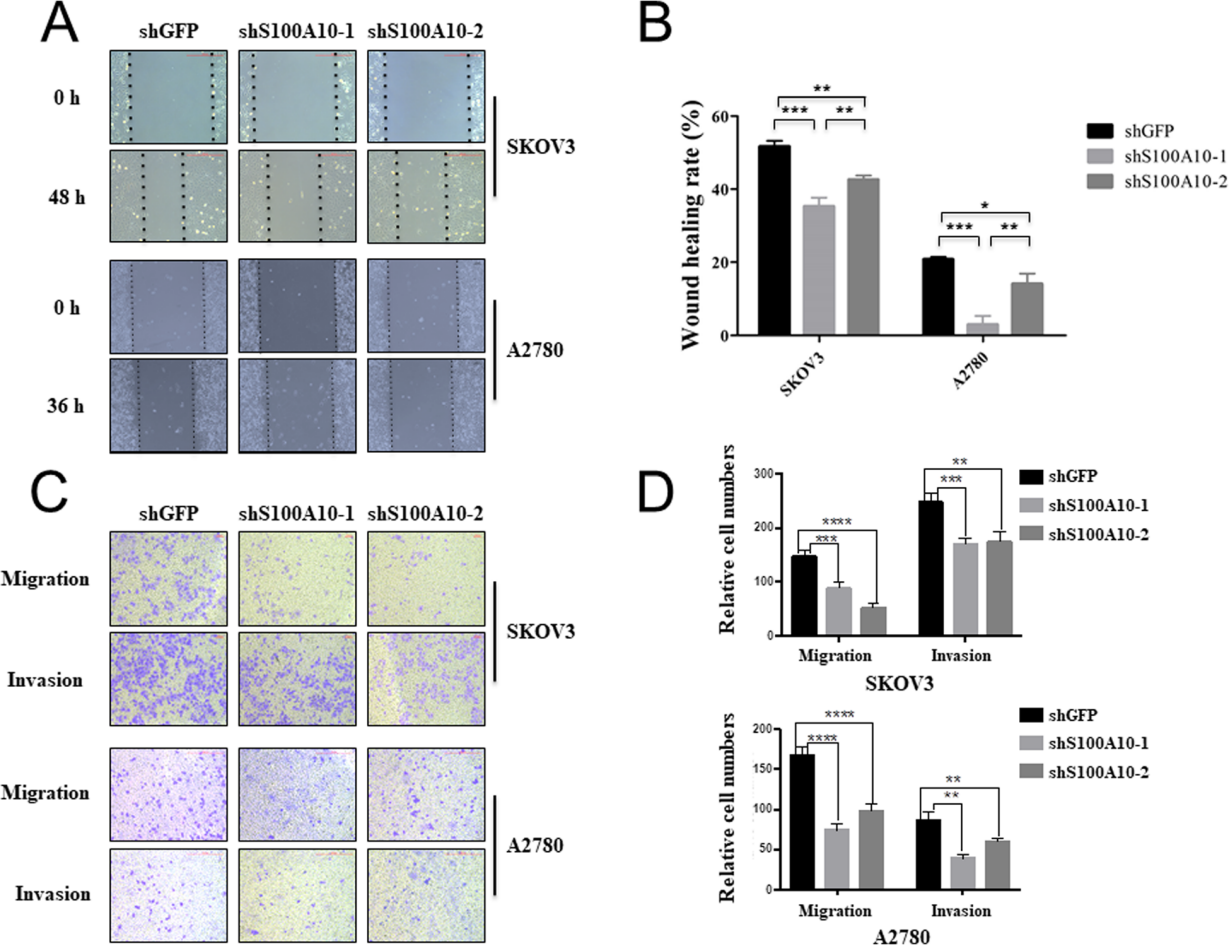
S100A10 knockdown inhibited cell migration and invasion. **a**, **b** Wound-healing analysis of cell migration in SKOV3 and A2780 cells transfected with shGFP, shS100A10–1 or shS100A10–2. **c**, **d** Transwell migration and invasion assay analyses in SKOV3 and A2780 cells transfected with shGFP, shS100A10–1 or shS100A10–2. Statistical significance was analyzed by ANOVA. Data are expressed as the mean ± standard deviation (*n* = 3 in each group)

To determine the effect of S100A10 on SKOV3 and A2780 migration and invasion, wound scratch and transwell assays were performed. It was found that SKOV3 and A2780 cells with S100A10 knockdown showed a slower closing of the scratch compared with the control group (Fig. 3a). Similar results were shown for cell migration and invasion. S100A10 knockdown remarkably inhibited cell migration and invasion abilities relative to the control group (Fig. 3b). These results demonstrate that S100A10 knockdown can significantly inhibit the metastatic properties of ovarian cancer cells.

### S100A10 suppression reduces chemoresistance

**Fig. 4:**
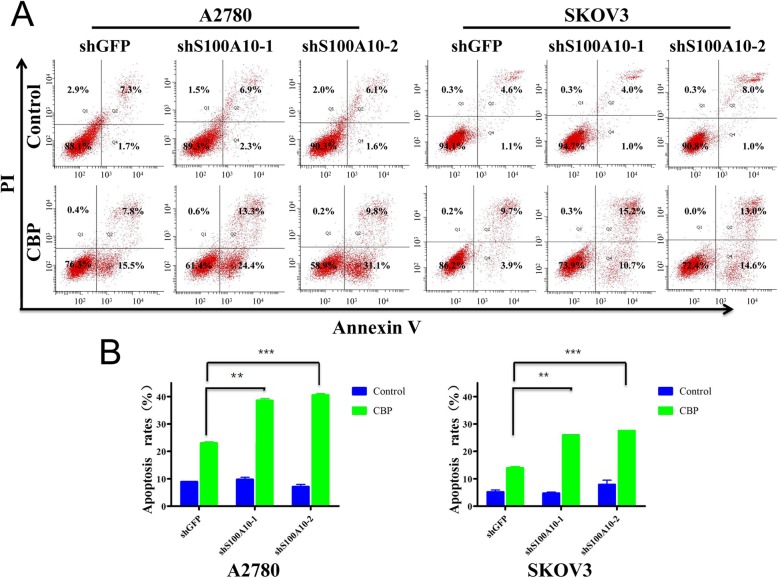
S100A10 mediates chemoresistance of A2780 and SKOV3 cells. **a** The apoptosis rate of A2780 and SKOV3 cells with or without carboplatin treatment as indicated was determined by flow cytometry. Cells were labeled with Annexin V -FITC & PI. **b** Bar chart of apoptosis ratios. Data are expressed as the mean ± standard deviation (*n* = 3 in each group)

**Fig. 5:**
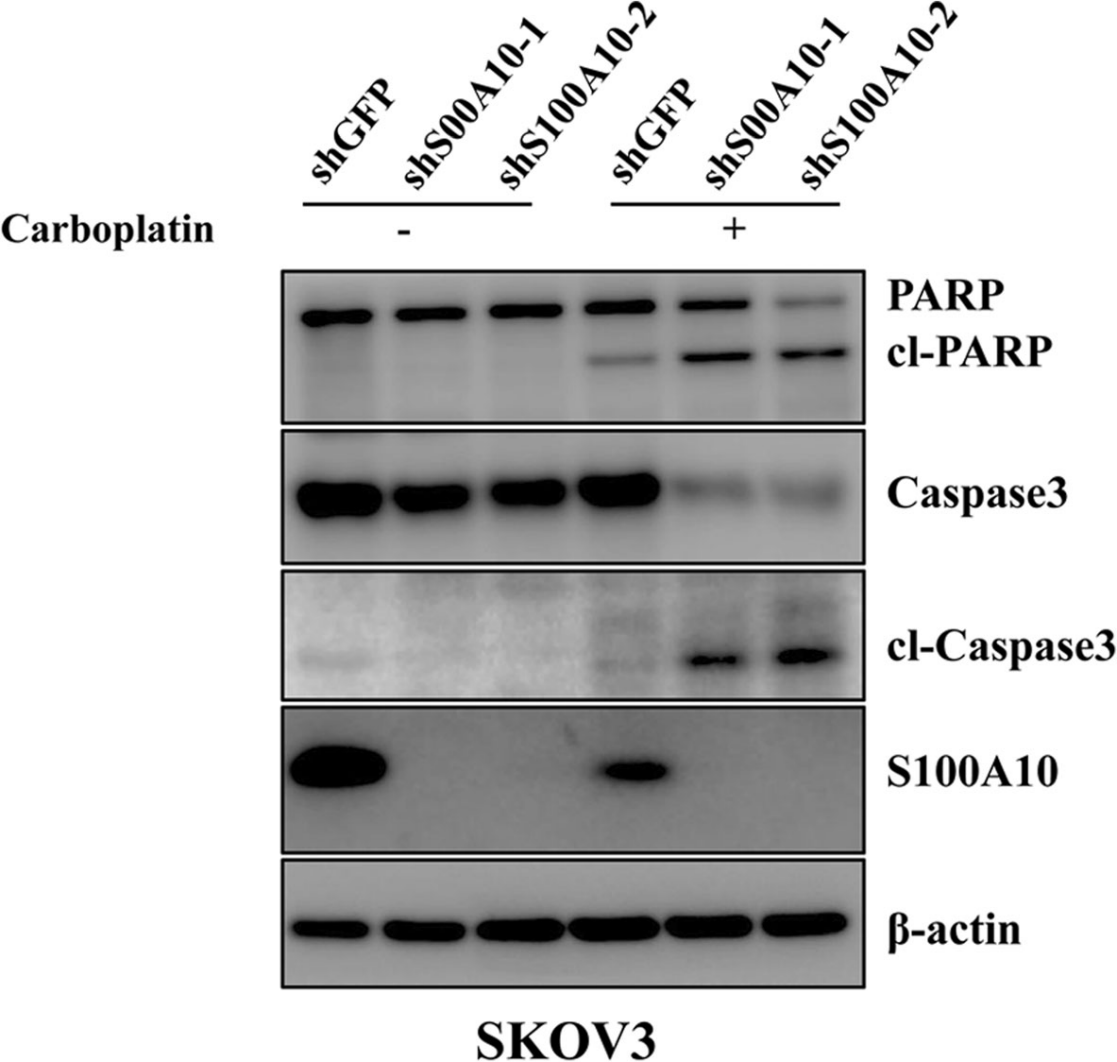
S100A10 mediates the sensitivity to carboplatin treatment. Western blot analysis of apoptosis-associated markers in SKOV3 cells treated with carboplatin. Data are expressed as the mean ± standard deviation (*n* = 3 in each group)

To investigate the impact of S100A10 on chemoresistance, Annexin V/PI staining and FACS were used to check the rate of apoptosis after carboplatin treatment. As shown in Fig. 4, S100A10 knockdown significantly increased the apoptosis rate in SKOV3 and A2780 cells after 24 h of carboplatin treatment, demonstrating that S100A10 promotes carboplatin resistance in ovarian cancer cells. Consistent with the effect of S100A10 on the apoptosis rate0, higher levels of the apoptotic markers cleaved PARP and cleaved Caspase3 were observed in S100A10-suppressed SKOV3 cells than in control cells (Fig. 5). These data indicated that S100A10 plays an important role in mediating sensitivity to carboplatin in ovarian cancer cells.

### Knockdown of S100A10 inhibited tumor growth in vivo

**Fig. 6:**
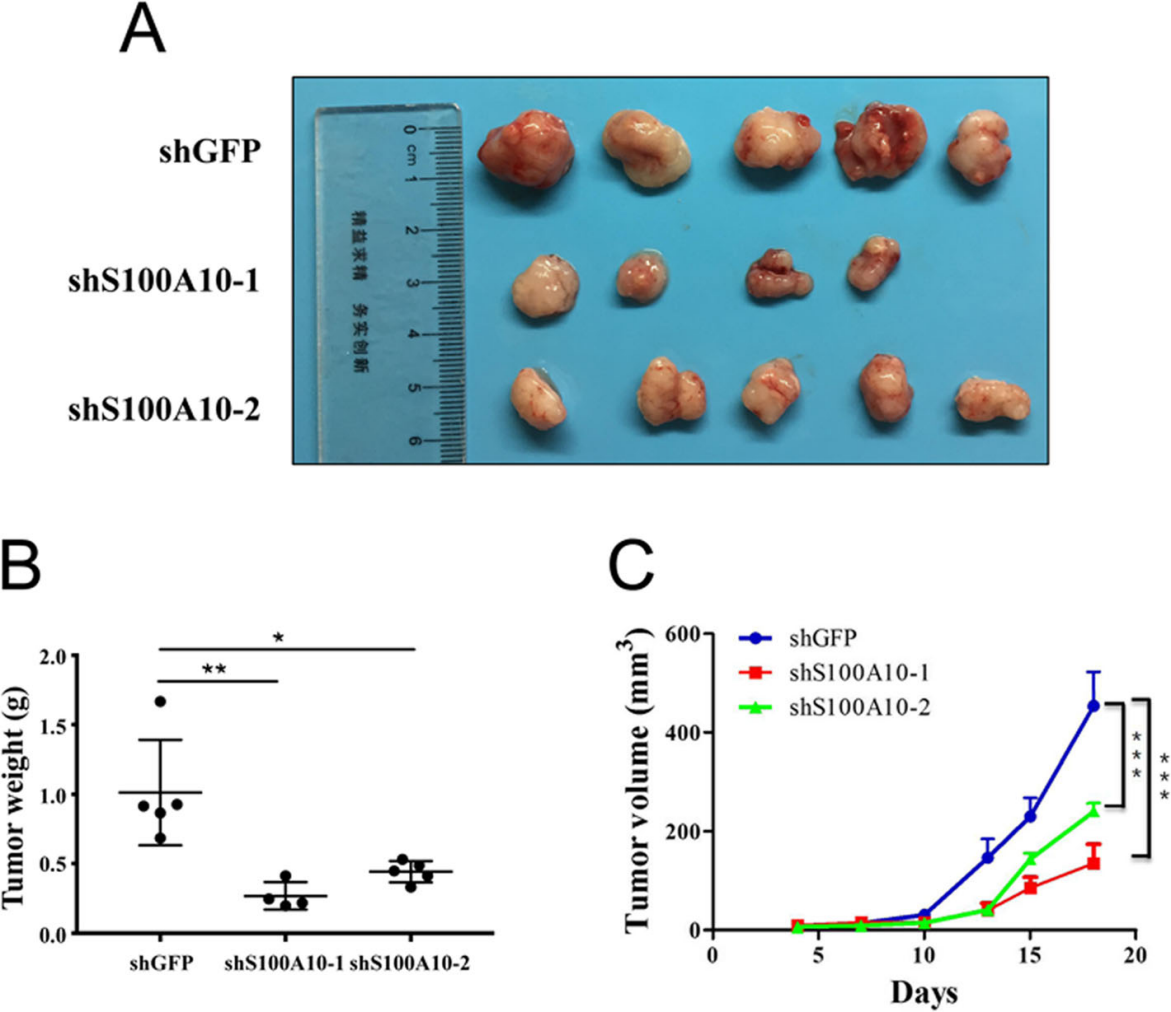
The effects of S100A10 knockdown on tumor growth in vivo. **a** shGFP, shS100A10–1 or shS100A10–2 were transfected into A2780 cells, which were then injected into NOD/SCID mice (*n* = 5). **b** Tumor weight was measured after sacrificing the NOD/SCID mice (*n* = 5). (**c**) Tumor volume of mice was measured after tumor inoculation. Statistical significance was analyzed by ANOVA. Data are expressed as the mean ± standard deviation (*n* = 3 in each group)

To further examine whether S100A10 was responsible for tumor growth in vivo, we subcutaneously injected A2780 cells transfected with shGFP, shS100A10–1 or shS100A10–2 into the flanks of NOD/SCID mice. Compared with the control, knockdown of S100A10 significantly inhibited tumor growth and showed a marked decrease in tumor weight (Fig. 6). In addition, it was found that tumor growth was slower in the shS100A10 group than in the shGFP group. Our results suggest that downregulation of S100A10 suppresses tumor growth in vivo.

## Discussion

Ovarian cancer is one of the most common gynecologic malignancies and often presents at an advanced stage. The absence of early symptoms and lack of reliable and specific early clinical diagnostic indicators have resulted in 75% of patients diagnosed at an advanced stage, a rate that has remained unchanged for several decades [17]. These poor clinical outcomes are largely due to the unsolved problems of metastasis, recurrence and the development of intrinsic or acquired drug resistance [17, 18]. Thus, a better understanding of the pathways underlying tumor progression and chemoresistance might aid in the design of more effective treatment strategies in ovarian cancer.

The S100 proteins are a group of small dimeric calcium-binding proteins with high structural homology and pleiotropic functions [19]. Abnormal expression of S100 proteins has been implicated in cancer [20, 21], autoimmune diseases [22], and chronic inflammatory disorders [23]. In particular, increased expression of S100A1 [24], S100A4 [25], S100A7 [26], and S100A14 [17] has been documented in multiple cancers including ovarian cancer. S100A1, A3, A6, A11, A13, A14, A16 and S100Z have been reported to be downregulated in oral squamous cell carcinoma (OSCC) patients compared with healthy controls. In contrast, S100A4, A8, A9, A10 and S100P have been reported to be overexpressed in OSCC patients compared with healthy controls [27]. S100A10, which contains two EF-binding motifs, is one of the members of the S100 protein family [28, 29]. Nevertheless, the association of S100A10 with clinicopathological characteristics in ovarian cancer and its biological functions have not yet been clarified. Our study is one of the most comprehensive studies of the expression of S100A10 in ovarian cancer. The present results clearly show that the upregulated expression of S100A10 in ovarian cancer is associated with carboplatin resistance, age at diagnosis and tumor grade. No significant association was observed between S100A10 expression and clinicopathological characteristics, including residual tumor size, level of CA125 in primary tumors and FIGO stage. The results reported here are partly consistent with the findings in lung adenocarcinomas, whereby Ken Katono et al. reported that high expression of S100A10 is significantly associated with poorer differentiation, a higher pathological TNM stage, more frequent and severe intratumoral vascular invasion and a poorer prognosis [11]. Additionally, in the present study, high expression levels of S100A10 predicted reduced PFS but not OS of ovarian cancer patients. Interestingly, this was not quantitatively consistent with the previous findings by Noor A. Lokman et al., in which S100A10 cytoplasmic expression was dramatically increased in ovarian cancer with reduced OS but not PFS [13]. This may be due to the limited sample size. We also found that the expression of S100A10 was detected in the cytoplasmic compartment of carboplatin-resistant ovarian cancer tissues. We understand that confirming the localization and expression of the S100A10 protein in normal tissues may better reveal the potential role of S100A10 in cancer cells. However, normal ovarian epithelium tissues were difficult to obtain. Therefore, we could not assess the role of the translocation of S100A10 on its biological function.

S100A10 has been suggested to be overexpressed in ocular surface squamous cell carcinoma and its expression level is associated with limbal epithelial cell proliferation and differentiation [30]. In a previous study, overexpression of miR-590-5p effectively decreased the expression of S100A10, which contributed to the suppression of HepG2 cell growth [31]. The results of the present study clearly demonstrate that S100A10 knockdown resulted in decreased proliferation and colony formation ability, increased apoptosis rate in vitro and inhibited tumor growth in vivo, advancing the current understanding of the involvement of S100A10 in tumorigenesis.

Previous studies have reported that the upregulation of S100A10 is associated with cancer metastasis. S100A10 accumulation, which is regulated by the succinyltransferase CPT1A and SIRT5-mediated desuccinylation, promotes gastric cancer cell invasion and migration [32]. Additionally, experimental studies have shown that S100A10, a recently identified binding partner of DLC1, regulates invasion of human lung cancer cells [33]. A recent study reported that S100A10 is required for tumor-promoting macrophage migration to tumor sites [34]. In the present study, the knockdown of S100A10 was found to inhibit the migration and invasion in human ovarian cancer cells. Furthermore, the wound-healing assay reconfirmed the impact of S100A10 on cell migration.

Regarding the significance of S100A10 expression in human colorectal cancer cells, Sayo Suzuki et al. reported that the upregulation of S100A10 showed significantly increased IC50 of L-OHP in COLO-320 CRC cells and downregulation of S100A10 in HT29 cells showed no apparent effect on sensitivity to L-OHP [7, 9]. The results of the present study demonstrated that the knockdown of S100A10 decreased resistance to carboplatin-based chemotherapy.

In conclusion, the present study provides evidence of a definitive role for S100A10 in the progression of ovarian cancer, and its expression levels may affect cell sensitivity to carboplatin. A xenograft study can better reveal the association between the expression level of S100A10 and carboplatin sensitivity. A xenograft study to investigate the sensitivity of SKOV3 cells to carboplatin will be considered in our further study. Additionally, the mechanisms of S100A10 involvement in carboplatin resistance are still unknown. Thus, further studies are required to fully elucidate the molecular mechanisms through which S100A10 acts as a potential biomarker of the response to carboplatin in ovarian cancer.

## Data Availability

All data generated or analyzed during this study are included in this published article or are available from the corresponding author on reasonable request.

## References

[1] Ricciardelli C, Lokman NA, Sabit I, Gunasegaran K, Bonner WM, Pyragius CE, Macpherson AM, Oehler MK (2018). Novel ex vivo ovarian cancer tissue explant assay for prediction of chemosensitivity and response to novel therapeutics. Cancer Lett.

[2] Hautaniemi S, Kozłowska E, Färkkilä A, Vallius T, Carpén O, Kemppainen J, Grénman S, Lehtonen R, Hynninen J, Hietanen S (2018). Mathematical modeling predicts response to chemotherapy and drug combinations in ovarian Cancer. Cancer Res.

[3] Chakraborty PK, Mustafi SB, Xiong X, Dwivedi SKD, Nesin V, Saha S, Zhang M, Dhanasekaran D, Jayaraman M, Mannel R, Moore K, McMeekin S, Yang D, Zuna R, Ding K, Tsiokas L, Bhattacharya R, Mukherjee P (2017). MICU1 drives glycolysis and chemoresistance in ovarian cancer. Nat Commun.

[4] Wendel J, Wang X, Hawkins S (2018). The Endometriotic tumor microenvironment in ovarian Cancer. Cancers (Basel).

[5] Ibrahim N, He L, Leong CO, Xing D, Karlan BY, Swisher EM, Rueda BR, Orsulic S, Ellisen W (2010). BRCA1-associated epigenetic regulation of p73 mediates an effector pathway for chemosensitivity in ovarian carcinoma. Cancer Res.

[6] O'Connell P, Surette A, Liwski R, Svenningsson P, Waisman D (2010). S100A10 regulates plasminogen-dependent macrophage invasion. Blood.

[7] Suzuki S, Tanigawara Y (2014). Forced expression of S100A10 reduces sensitivity to oxaliplatin in colorectal cancer cells. Proteome Sci.

[8] Hedhli N, Falcone DJ, Huang B, Cesarman-Maus G, Kraemer R, Zhai H, Tsirka SE, Santambrogio L, Hajjar KA (2012). The annexin A2/S100A10 system in health and disease: emerging paradigms. J Biomed Biotechnol.

[9] Suzuki S, Yamayoshi Y, Nishimuta A, Tanigawara Y (2011). S100A10 protein expression is associated with oxaliplatin sensitivity in human colorectal cancer cells. Proteome Sci.

[10] McKiernan E, McDermott E, Evoy D, Crown J, Duffy M (2011). The role of S100 genes in breast cancer progression. Tumour Biol.

[11] Katono K, Sato Y, Jiang SX, Kobayashi M, Saito K, Nagashio R, Ryuge S, Satoh Y, Saegusa M, Masuda N (2016). Clinicopathological significance of S100A10 expression in lung adenocarcinomas. Asian Pac J Cancer Prev.

[12] El-Rifai W, Moskaluk C, Abdrabbo M (2002). Gastric cancers overexpress S100A calcium-binding proteins. Cancer Res.

[13] Bydoun M, Sterea A, Liptay H, Uzans A, Huang WY, Rodrigues GJ, Weaver ICG, Gu H, Waisman DM (2018). S100A10, a novel biomarker in pancreatic ductal adenocarcinoma. Mol Oncol.

[14] Lokman NA, Pyragius CE, Ruszkiewicz A, Oehler MK, Ricciardelli C (2016). Annexin A2 and S100A10 are independent predictors of serous ovarian cancer outcome. Transl Res.

[15] Li Y, Kong Y, Zhou Z, Chen H, Wang Z, Hsieh YC, Zhao D, Zhi X, Huang J, Zhang J, Li H, Chen C (2013). The HECTD3 E3 ubiquitin ligase facilitates cancer cell survival by promoting K63-linked polyubiquitination of caspase-8. Cell Death Dis.

[16] Shu T, Li Y, Wu X, Li B, Liu Z (2017). Down-regulation of HECTD3 by HER2 inhibition makes serous ovarian cancer cells sensitive to platinum treatment. Cancer Lett.

[17] Qian J, Ding F, Luo A, Liu Z, Cui Z (2016). Overexpression of S100A14 in human serous ovarian carcinoma. Oncol Lett.

[18] Yang T, Cheng J, You J, Yan B, Liu H, Li F (2018). S100B promotes chemoresistance in ovarian cancer stem cells by regulating p53. Oncol Rep.

[19] Bresnick AR (2018). S100 proteins as therapeutic targets. Biophys Rev.

[20] Tong L, Lan W, Lim RR, Chaurasia SS (2014). S100A proteins as molecular targets in the ocular surface inflammatory diseases. Ocul Surf.

[21] Brenner AK, Bruserud Ø (2018). S100 Proteins in Acute Myeloid Leukemia. Neoplasia.

[22] Manolakis AC, Kapsoritakis AN, Tiaka EK, Potamianos SP (2011). Calprotectin, calgranulin C, and other members of the s100 protein family in inflammatory bowel disease. Dig Dis Sci.

[23] Yammani RR (2012). S100 proteins in cartilage: role in arthritis. Biochim Biophys Acta.

[24] DeRycke MS, Andersen JD, Harrington KM, Pambuccian SE, Kalloger SE, Boylan KL, Argenta PA, Skubitz AP (2009). Am J Clin Pathol.

[25] Lv Y, Niu Z, Guo X, Yuan F, Liu Y (2018). Serum S100 calcium binding protein A4 (S100A4, metatasin) as a diagnostic and prognostic biomarker in epithelial ovarian cancer. Br J Biomed Sci.

[26] Lin M, Xia B, Qin L, Chen H, Lou G (2018). S100A7 regulates ovarian Cancer cell metastasis and Chemoresistance through MAPK signaling and is targeted by miR-330-5p. DNA Cell Biol.

[27] Raffat MA, Hadi NI, Hosein M, Mirza S, Ikram S, Akram Z (2018). S100 proteins in oral squamous cell carcinoma. Clin Chim Acta.

[28] Holthenrich A, Gerke V (2018). Regulation of von-Willebrand Factor Secretion from Endothelial Cells by the Annexin A2-S100A10 Complex. Int J Mol Sci.

[29] Bydoun M, Waisman DM (2014). On the contribution of S100A10 and annexin A2 to plasminogen activation and oncogenesis: an enduring ambiguity. Future Oncol.

[30] Li J, Riau AK, Setiawan M, Mehta JS, Ti SE, Tong L, Tan DT, Beuerman RW (2011). S100A expression in normal corneal-limbal epithelial cells and ocular surface squamous cell carcinoma tissue. Mol Vis.

[31] Shan X, Miao Y, Fan R, Qian H, Chen P, Liu H, Yan X, Li J, Zhou F (2013). MiR-590-5P inhibits growth of HepG2 cells via decrease of S100A10 expression and inhibition of the Wnt pathway. Int J Mol Sci.

[32] Wang Chao, Zhang Chen, Li Xiang, Shen Jiajia, Xu Yue, Shi Hui, Mu Xianmin, Pan Jinshun, Zhao Ting, Li Mengjing, Geng Biao, Xu Che, Wen Hao, You Qiang (2018). CPT1A-mediated succinylation of S100A10 increases human gastric cancer invasion. Journal of Cellular and Molecular Medicine.

[33] Yang X, Popescu N, Zimonjic D (2011). DLC1 interaction with S100A10 mediates inhibition of in vitro cell invasion and tumorigenicity of lung cancer cells through a RhoGAP-independent mechanism. Cancer Res.

[34] Phipps K, Surette A, O'Connell P, Waisman D (2011). Plasminogen receptor S100A10 is essential for the migration of tumor-promoting macrophages into tumor sites. Cancer Res.

